# PgZHD18‐PgWDR5b increases basal thermotolerance in *Physalis grisea* by lignin biosynthesis dependent on H3K4me3

**DOI:** 10.1111/tpj.71131

**Published:** 2026-09-21

**Authors:** Hong Li, Guanzhuo Kong, Yihan Zhang, Yan Ren, Jiayin Niu, Xiaochun Zhao, Qiaofang Shi, Yihe Yu

**Affiliations:** ^1^ College of Horticulture and Plant Protection Henan University of Science and Technology Luoyang Henan 471023 China; ^2^ Henan Provincial Engineering Research Center on Characteristic Berry Germplasm Innovation & Utilization Luoyang Henan China

**Keywords:** *Physalis grisea*, high temperature stress, ZHD, TrxG, H3K4me3

## Abstract

*Physalis grisea* is a widely valued orphan crop with medicinal properties and edible functional fruits. Under global warming, high temperature stress (HTS) severely restricts the growth and development of *P. grisea*, while the epigenetic regulatory mechanism underlying its HTS response remains largely unclear. In this study, we identified the ZINC FINGER HOMEODOMAIN (ZHD) transcription factor PgZHD18 as a positive regulator of basal thermotolerance in *P. grisea*. Functional assays showed that *PgZHD18* enhances basal thermotolerance by improving ROS scavenging capacity, alleviating membrane damage, and maintaining chlorophyll stability. Further molecular verification revealed that PgZHD18 directly binds to the promoters of key lignin biosynthetic genes (*PgPAL1*, *PgC3H1*, *PgC4H*, *PgCAD3*) and activates their transcription, thus promoting lignin accumulation. Moreover, PgZHD18 interacts with the Trithorax Group (TrxG) protein PgWDR5b, which increases H3K4me3 deposition at the promoters of lignin biosynthetic genes and further amplifies their transcriptional activation, synergistically enhancing basal thermotolerance. Collectively, our findings reveal a dual regulatory module of transcriptional activation coupled with epigenetic modification in plant heat response, and provide a theoretical basis and molecular targets for breeding heat‐resistant crop varieties.

## Significance Statement

This study uncovers a novel epigenetic regulatory module in which PgZHD18 interacts with PgWDR5b to enhance lignin biosynthesis via H3K4me3 modification, thereby promoting basal thermotolerance in *Physalis grisea*. Our findings provide new insights into how plants cope with heat stress and offer potential molecular targets for breeding stress‐resistant crops.

## INTRODUCTION

Climate change‐induced extreme heat events pose a serious threat to plants survival and agricultural productivity (Lesk et al., 2022; Rivero et al., 2022; Verma et al., 2025). High temperature stress (HTS) disrupts normal physiological processes and inhibits plants growth and development, leading to a range of adverse effects including damage to the photosynthetic apparatus, membrane lipid peroxidation, and protein denaturation (Saidi et al., 2011; Sato et al., 2024; Wu et al., 2024). To cope with HTS, plants have evolved sophisticated defense mechanisms, such as activating antioxidant systems, inducing HEAT SHOCK PROTEIN (HSP), and reprogramming metabolic pathways (He et al., 2025; Shekhawat et al., 2022; Zhang et al., 2025).

The perception of HTS and subsequent physiological adaptations rely on precise and coordinated regulation by various transcription factors (TFs) (Bakery et al., 2024; Kan et al., 2023; Mou et al., 2024). Under mild high temperatures (28–32°C), plants trigger a thermo‐morphogenetic response characterized by hypocotyl elongation and leaf hyponasty to improve heat dissipation. However, under lethal extreme high temperature stress (42°C in this study), thermo‐morphogenetic is typically suppressed, and plants activate basal thermotolerance pathways to ensure survival. Under HTS, TFs play central roles in modulating growth and developmental patterns to improve acclimation (Ding et al., 2020). HEAT SHOCK FACTORs (HSFs) are key regulators of the HTS response. They bind to HEAT SHOCK ELEMENTs (HSEs) and activate the expression of HSPs, thereby protecting proteins from thermal damage (Guo et al., 2020; Zhang et al., 2025). In rice, OsHSFA4d, as a core transcription factor regulating stress responses, coordinately controls rice thermotolerance and disease resistance through a phosphorylation switch (Fang et al., 2025). ZxWRKY4 from *Zygophyllum xanthoxylum* enhances thermotolerance by activating heat‐related genes such as *HSFA2*, *HSP70*, and *DREB2C* (Wang, Wang, et al., 2025). Furthermore, other TFs also contribute to heat tolerance through two major pathways closely related to plant stress resistance: antioxidant defense system and cell wall modification. Furthermore, bZIP, NAC, and MYB type TFs also contribute to the coordination of heat responses primarily through regulating genes involved in antioxidant defense, osmolyte biosynthesis, and cell wall modification (Das et al., 2019; Liu et al., 2023; Xiong et al., 2025). CibZIP43 in *Carya illinoinensis* enhances thermotolerance by improving chlorophyll retention, water content, ROS scavenging, antioxidant enzyme activities, and membrane stability (Wang, Jiang, et al., 2025). DcNAC41 in carnation (*Dianthus caryophyllus* L.) protects against HTS by increasing ROS scavenging capacity. (Zhao et al., 2025). SlMYB41 can effectively reduce the damage caused by HTS by protecting cell membrane integrity in *Solanum lycopersicum* (Wang et al., 2023).

In response to environmental stresses, histone modifications mediated by epigenetic mechanisms play a crucial role in the regulation of gene expression in plants (Chai et al., 2024; Liu et al., 2022; Ueda & Seki, 2020). Among these, trimethylation of lysine 4 on histone H3 (H3K4me3) serves as a key transcriptional activation mark that facilitates gene transcription by dynamically modulating chromatin structure (Perrella et al., 2022; Shi et al., 2024; Wang, He, et al., 2025). Studies have shown that HTS can induce the deposition of H3K4me3 modifications around transcriptional start sites, leading to the activation of heat‐responsive genes (Kim et al., 2015; Liu et al., 2015). WD40 repeat protein WDR5b, a core component of the COMPASS‐like complex, plays a critical role in H3K4me3 catalysis by recognizing histone substrates and recruiting methyltransferases (Jiang et al., 2011; Wang et al., 2022). Emerging evidence indicates that certain TFs interact with epigenetic regulators such as WDR5b to recruit H3K4me3 modifications to specific genomic loci, thereby regulating stress‐responsive gene expression (Kong et al., 2025; Song et al., 2015).

Lignin, a major component of the plants secondary cell wall, plays a critical role in abiotic stress resistance, especially under HTS, by acting as a physical barrier and enhancing structural integrity (Han et al., 2022; Yang et al., 2025). Thickening of the cell wall and increased lignification help maintain cellular integrity, reduce water loss, and improve mechanical strength, thereby contributing to thermotolerance (Han et al., 2022; Hayford et al., 2022). Furthermore, lignin is involved in plants defense responses and may contribute to scavenging or reducing the production of reactive ROS. The biosynthesis of lignin is tightly regulated through a multistep enzymatic pathway involving key enzymes such as phenylalanine ammonia‐lyase (PAL), cinnamate‐4‐hydroxylase (C4H), 4‐coumarate: CoA ligase (4CL), and peroxidase (POD) (Whetten & Sederoff, 1995; Yoon et al., 2015). For example, the increase of meso‐lignin content in apple (*Malus domestica*) leaves under high temperature synergizes with the plants' oxidative stress response mechanism, thereby enhancing its basal heat tolerance (Qiu et al., 2026). Recent studies have revealed that the expression of key genes in the lignin biosynthesis is subject to epigenetic regulation, notably through H3K4me3 modification (Fal et al., 2023; Ma et al., 2025).

*P. grisea*, a medicinally and economically important solanaceous plant, is valued for its fruits rich in vitamin C, carotenoids, phenolic compounds, and various alkaloids, which demonstrate significant antioxidant, anti‐inflammatory, and immunomodulatory activities (Dale et al., 2024; Valdivia‐Mares et al., 2016). These properties underscore its potential for use in functional foods and modernized herbal medicine (Jassim et al., 2024). However, under HTS, the seedlings of *P. grisea* show impaired development, leading to fruit abortion and premature senescence or mortality of the plants. (Jing et al., 2025; Kong et al., 2025). Additionally, HTS leads to a marked reduction in secondary metabolite content, thereby diminishing its medicinal value. This study focuses on the PgZHD18‐PgWDR5b module which regulates the transcriptional activity of key lignin biosynthetic genes through modulating H3K4me3 deposition in response to HTS in *P. grisea*. Our findings reveal not only a synergistic interaction between epigenetic modifications, TFs and metabolic pathways under HTS, but also provide a molecular basis for the production of basal thermotolerance in *P. grisea* varieties.

## RESULTS

### Identification and expression analysis of PgZHD18

**Figure 1:**
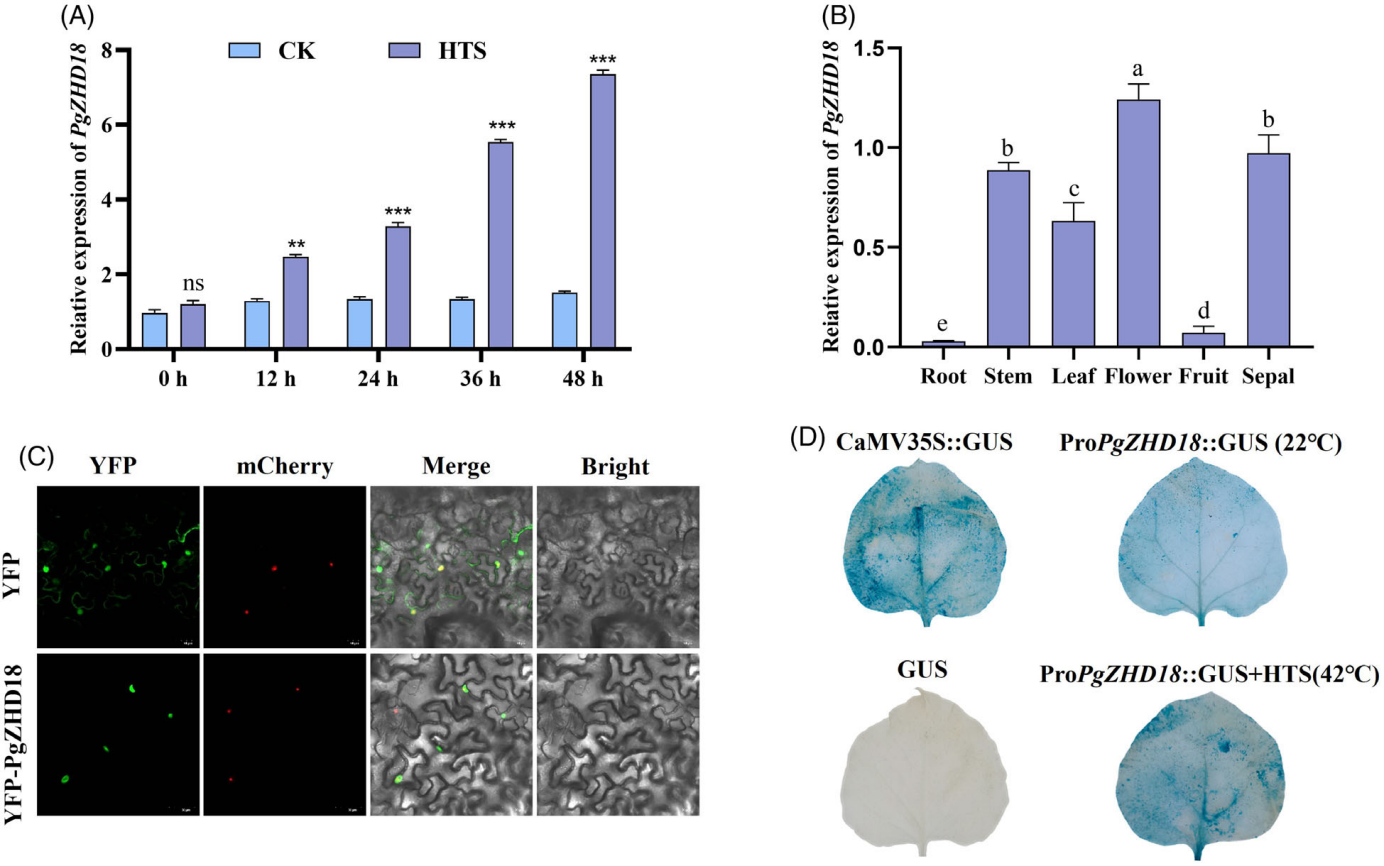
Expression pattern and functional localization of *PgZHD18*. (A) *PgZHD18* expression levels at 0 h, 12 h, 24 h, 36 h, and 48 h of 42°C HTS in total plants. (B) Expression levels of *PgZHD18* in different tissues of *P. grisea*. The data are presented as means ±SDs (*n* = 3). Significant differences were analyzed by one‐way ANOVA followed by Duncan's multiple range test (*P* < 0.05). (C) Subcellular localization of PgZHD18 in *Nicotiana benthamiana* leaves. Scale bar = 50 μm. The YFP‐PgZHD18 fusion protein was localized in the nucleus of *N. benthamiana* cells. YFP was used as a control. The nucleus was labeled with red fluorescence (mCherry), and YFP fluorescence was green. The Bright field is the normal view, and the merge is a composite image of three images. (D) GUS chemical tissue staining results. CaMV35S::GUS was used as a positive control. GUS and Pro*PgZHD18*::GUS (22°C) treated at 22°C for 24 h were used as negative controls. Pro*PgZHD18*::GUS + HTS (42°C) treated at 42°C for 24 h were used as experimental group. Darker staining represents higher activation of the promoter. Asterisks indicate statistically significant differences (**, *P* < 0.01; ***, *P* < 0.001; ns, not significant, Student's *t*‐test). Three biological replicates were set for each assay.

PgZHD18 as a member of the ZHD TFs family was identified in the *P. grisea* genome. Transcriptome analysis under HTS revealed that the expression of *PgZHD18* (*Phygri12g016660*) was strongly induced by high temperature (Figure S1). Analysis of the expression pattern of *PgZHD18* by real‐time quantitative PCR (RT‐qPCR) detected a significant upregulation of *PgZHD18* expression after HTS treatment (Figure 1A). Further investigation of the tissue‐specific expression pattern of *PgZHD18* showed that its transcript level was highest in flowers, followed by stems and sepal. It was lowest in roots (Figure 1B). Subcellular localization assays confirmed that PgZHD18 is localized to the nucleus (Figure 1C), consistent with its predicted function as a TF. Promoter activity assays indicated that the *PgZHD18* promoter can be activated by high temperature (Figure 1D). These results demonstrate that PgZHD18 was specifically activated by HTS and plays a transcriptional regulatory role in the nucleus.

### PgZHD18 positively regulates basal thermotolerance in *P. grisea*

**Figure 2:**
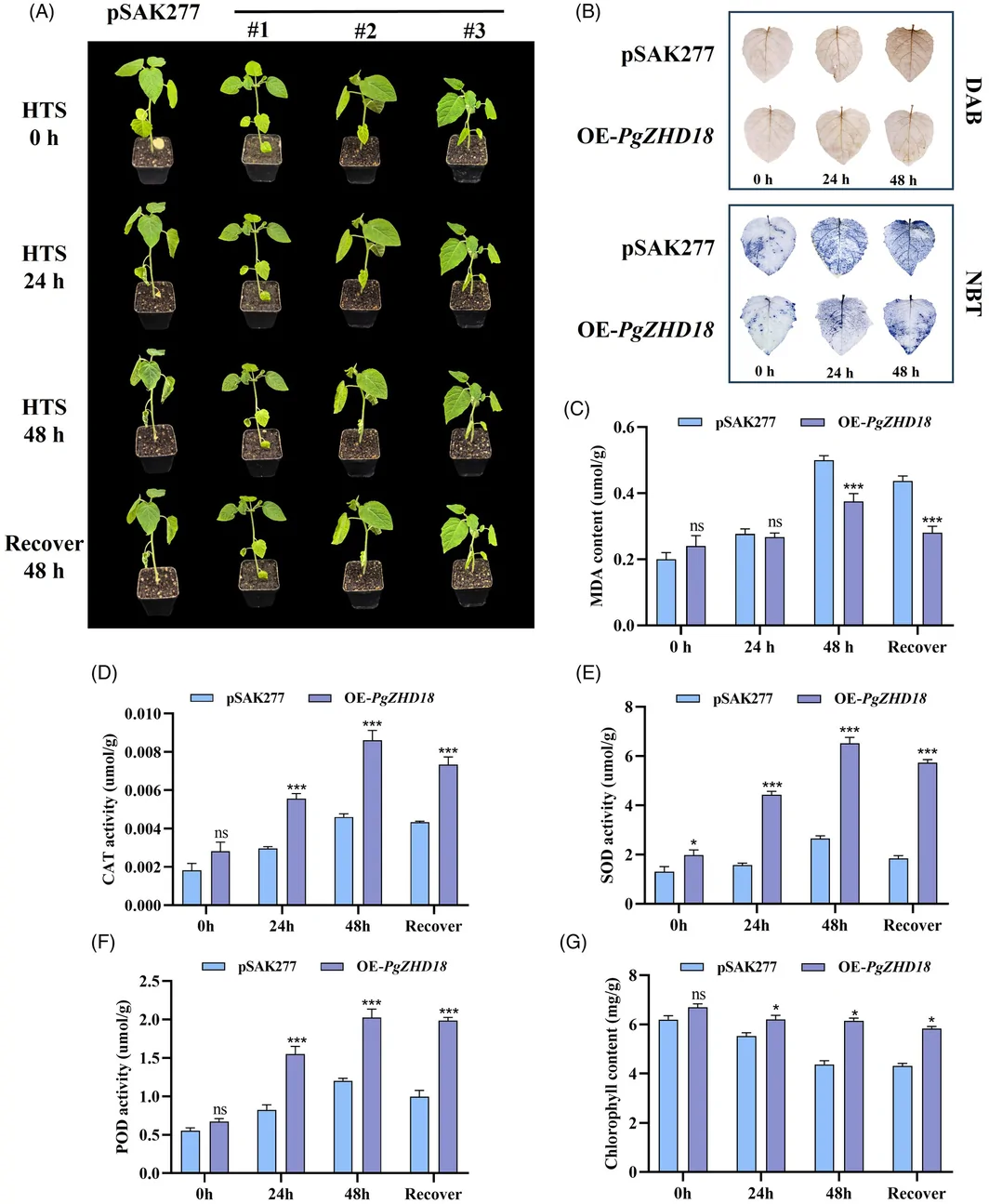
Overexpression of *PgZHD18* enhances thermotolerance in *P. grisea*. (A) Thermotolerant phenotype of *PgZHD18* overexpressed plants. Three independent OE‐*PgZHD18* lines (#1, #2, #3) and control (pSAK277) plants were grown in pots and treated at 42°C for 48 h at six true leaf stages followed by recovery at 22°C for 48 h. (B) Oxidative damage to *P. grisea* leaves induced by HTS. Detection of H_2_O_2_ and O_2_^−^ by DAB and NBT chemical staining. (C–G) MDA, CAT, SOD, POD and chlorophyll contents in OE‐*PgZHD18* plants. Assays were performed at 0 h, 24 h, 48 h of treatment at 42°C and 48 h of recovery at 22°C. Each line contained 20 plants, and the experiment was repeated three times independently. Asterisks indicate statistically significant differences (*, *P* < 0.05; ***, *P* < 0.001; ns, not significant, Student's *t*‐test). Three biological replicates were set for each assay.

**Figure 3:**
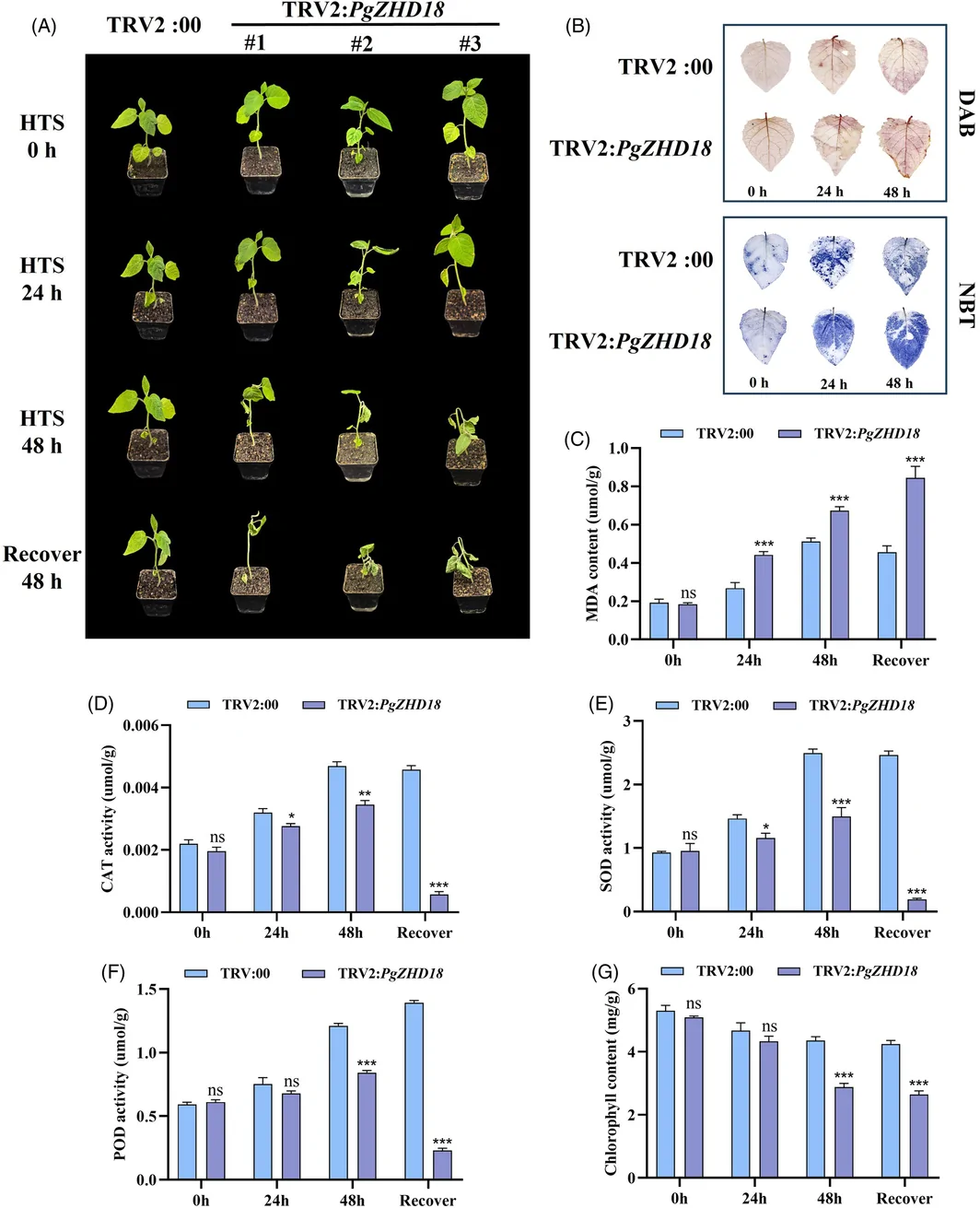
Silence of *PgZHD18* enhances heat sensitivity in *P. grisea*. (A) Thermosensitive phenotype of *PgZHD18* silenced plants. Three independent TRV2:*PgZHD18* lines (#1, #2, #3) and control (TRV2:00) plants were grown in pots and treated at 42°C for 48 h at six true leaf stages followed by recovery at 22°C for 48 h. (B) Oxidative damage to *P. grisea* leaves induced by HTS. Detection of H_2_O_2_ and O_2_^−^ by DAB and NBT chemical staining. (C–G) MDA, CAT, SOD, POD, and chlorophyll contents in TRV2:*PgZHD18* plants. Assays were performed at 0 h, 24 h, 48 h of treatment at 42°C, and 48 h of recovery at 22°C. Each line contained 20 plants, and the experiment was repeated three times independently. Asterisks indicate statistically significant differences (*, *P* < 0.05; **, *P* < 0.01; ***, *P* < 0.001; ns, not significant, Student's *t*‐test). Three biological replicates were set for each assay.

To investigate the function of *PgZHD18* under HTS, *PgZHD18* overexpressing transgenic lines (OE‐*PgZHD18*) were generated (Figure S2A,B). After 48 h of 42°C heat stress treatment, pSAK277 control plants showed severe leaf wilting, yellowing and necrosis, while all three OE‐*PgZHD18* lines maintained green leaves and upright stems. After 48 h of recovery at 22°C, most control plants died, while OE‐*PgZHD18* plants resumed normal growth (Figure 2A). Histochemical staining with nitroblue tetrazolium (NBT) and 3,3′‐diaminobenzidine (DAB) revealed lower accumulation of superoxide anions and hydrogen peroxide in OE‐*PgZHD18* leaves than in WT under HTS (Figure 2B). In addition, OE‐*PgZHD18* plants showed a decrease in Malondialdehyde (MDA) levels, increased antioxidant enzymes (Catalase, CAT, Superoxide Dismutase, SOD and Peroxidase, POD) activity, and an increase in chlorophyll levels (Figure 2D–H). Survival rate statistics showed that all three independent OE‐*PgZHD18* lines had significantly higher survival rates than the control after HTS treatment and recovery (Figure S2C). In contrast, TRV2:*PgZHD18* plants generated by VIGS (Figure S3A,B) showed increased sensitivity to HTS. After 24 h of treatment, TRV2:*PgZHD18* plants exhibited severe stress‐induced phenotypes, including leaf yellowing and wilting, reduced stem mechanical strength, and heat‐induced stem twisting and lodging. After 48 h of HTS treatment, TRV2:*PgZHD18* plants almost completely wilted and died, and no improvement was observed even after 48 h of recovery at 22°C (Figure 3A). Stronger NBT and DAB staining was observed in TRV2:*PgZHD18* leaves compared to both WT and OE‐*PgZHD18* plants after HTS (Figure 3B). Moreover, the silenced plants had a higher MDA content and reduced CAT, SOD, and POD activities and decreased chlorophyll levels at both 24 h and 48 h of HTS treatment (Figure 3D–H). Survival rate statistics confirmed that all three independent TRV2:*PgZHD18* lines had significantly lower survival rates than the control (Figure S3C). These results demonstrate that PgZHD18 positively regulates basal thermotolerance in *P. grisea* by playing a key role in alleviating HTS induced oxidative damage.

To directly investigate whether lignin biosynthesis is induced by HTS and whether this response is regulated by *PgZHD18*, we measured lignin content in all genotypes before and after 48 h of 42°C HTS treatment (Figures S2D and 3D). In control plants, lignin content was increased by 18–25% after HTS treatment, confirming that in this species, the increase in lignin biosynthesis is a physiological response to the HTS treatment to which it was subjected. Under both normal conditions and HTS, OE‐*PgZHD18* plants had significantly higher lignin content than the pSAK277 control, while TRV2:*PgZHD18* plants had significantly lower lignin content than the TRV2:00 control. The lignin content of OE‐*PgZHD18* increased by 47–55% after HTS treatment, while that of TRV2:*PgZHD18* increased by only 8–15%. In addition, lignin degradation in TRV2:*PgZHD18* plants died after HTS treatment, resulting in a decrease in lignin content after 48 h of recovery culture, indicating that *PgZHD18* silencing severely impaired the ability of plants to accumulate and maintain lignin under high temperature stress. These results suggest that *PgZHD18* positively regulates lignin biosynthesis under both normal and high temperature stress, and the elevated lignin content is associated with the improved survival rate and reduced stem lodging phenotype of plants under HTS.

### PgZHD18 regulates stem development and lignin biosynthesis in *P. grisea*

**Figure 4:**
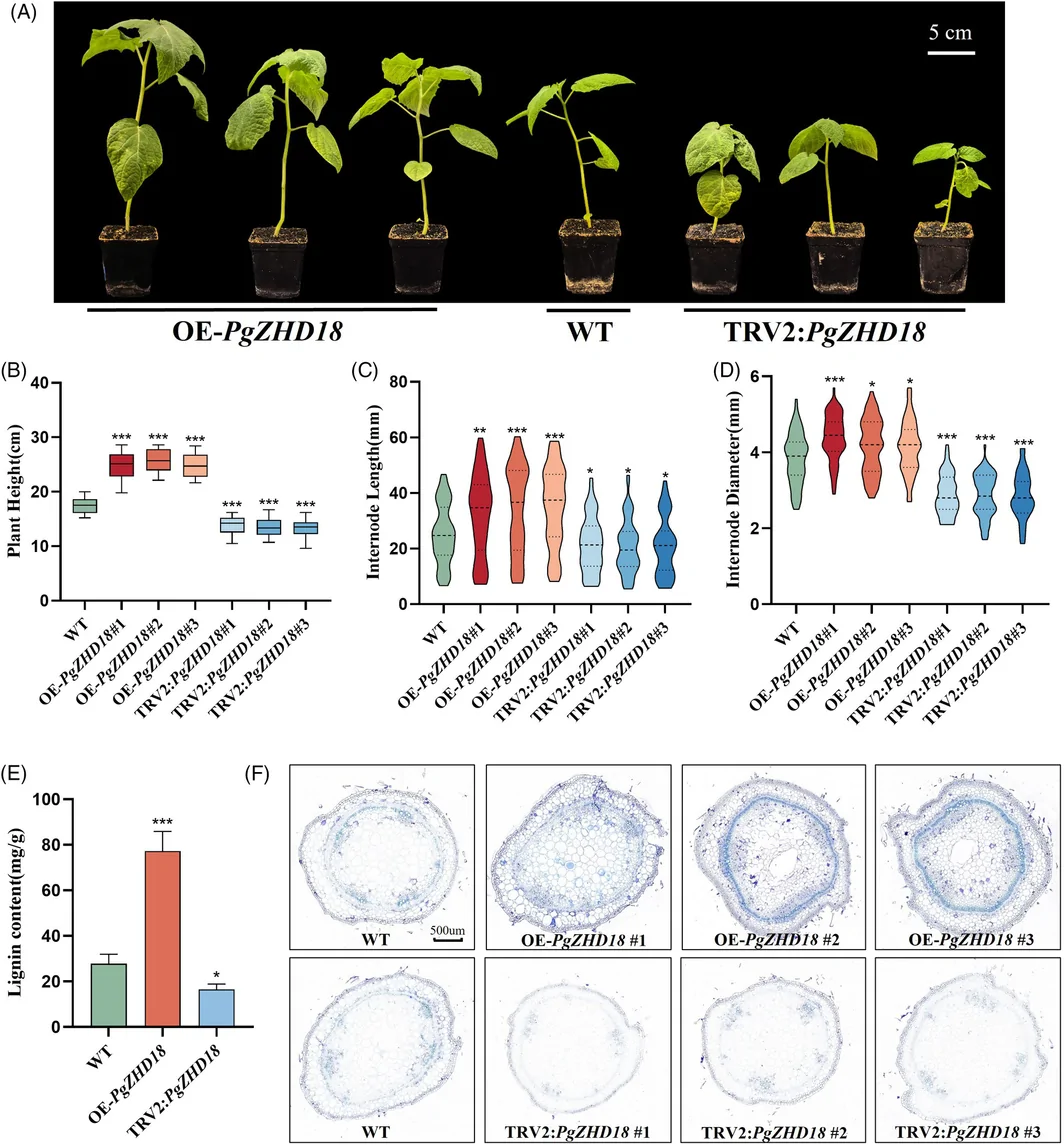
*PgZHD18* regulates the growth and lignin biosynthesis in *P. grisea*. (A) Growth phenotypes of OE‐*PgZHD18*, TRV2:*PgZHD18* and wild‐type (WT) plants at 45‐day stage under normal conditions. Scale bar = 5 cm. (B–D) Statistical analysis of phenotypic characteristics: plant height, internode length, and internode diameter in transgenic plants. (E) Lignin content in OE‐*PgZHD18*, TRV2:*PgZHD18*, and WT plants. (F) Under normal conditions, the lignin staining of the stems in OE‐*PgZHD18*, TRV2:*PgZHD18*, and WT plants. Lignin staining appears blue. Scale bar = 500 μm. Data were obtained from three independent biological experiments, with 20 plants per line in each experiment. Asterisks indicate statistically significant differences (*, *P* < 0.05; **, *P* < 0.01; ***, *P* < 0.001; ns, not significant, Student's *t*‐test). Three biological replicates were set for each assay.

Phenotypic comparison between OE‐*PgZHD18* and TRV2:*PgZHD18* plants revealed distinct stem developmental patterns at the same growth stage (45 days) under normal conditions (Figure 4A). *OE‐PgZHD18* plants displayed increased plant height, longer internodes, and thicker stems, whereas TRV2:*PgZHD18* plants exhibited reduced height, shorter internodes, and weaker stems (Figure 4B–D). These data were obtained from three independent biological experiments, with 20 plants per line in each experiment, and showed high reproducibility with consistent trends across all experiments. We hypothesized that these phenotypic differences might be associated with variations in lignin content. To verify this hypothesis, we measured lignin content and observed lignin deposition in stem cross sections. Lignin levels were measured for all genotypes and cross sections of the stem of the third internode of the 45 days were examined. Lignin content was significantly higher in OE‐*PgZHD18* plants compared to TRV2:*PgZHD18* plants (Figure 4E). Consistent with this, histological staining revealed stronger lignin deposition in OE‐*PgZHD18* stems and reduced lignification in TRV2:*PgZHD18* stems (Figure 4F). These results suggested that *PgZHD18* positively regulates lignin biosynthesis under normal conditions, which may promote stem thickening and mechanical strength to withstand HTS.

**Figure 5:**
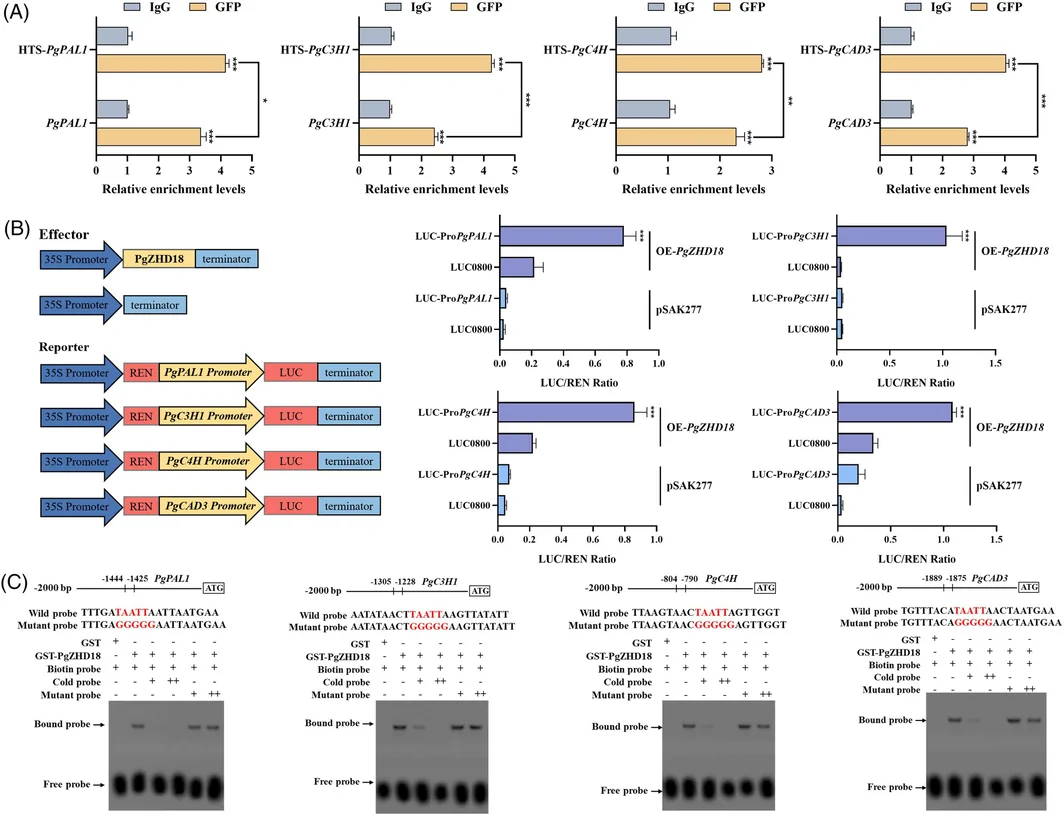
PgZHD18 directly binds and regulates the expression of *PgPAL1*, *PgC4H*, *PgC3H1*, and *PgCAD3*. (A) ChIP‐qPCR validation of *PgPAL1*, *PgC4H*, *PgC3H1*, and *PgCAD3* enrichment levels in OE‐*PgZHD18* plants at normal temperature (22°C) and high temperature (42°C). The enrichment levels of *PgPAL1*, *PgC4H*, *PgC3H1*, and *PgCAD3* were significantly enhanced under HTS. (B) Schematic diagram of dual luciferase vector construction. Analysis of LUC/REN ratio in the dual luciferase assay. (C) EMSA of PgZHD18 interacting with *PgPAL1*, *PgC4H*, *PgC3H1*, and *PgCAD3* via the TAATT cis‐element. Asterisks indicate statistically significant differences (*, *P* < 0.05; **, *P* < 0.01; ***, *P* < 0.001; ns, not significant, Student's *t*‐test). Three biological replicates were set for each assay.

To further dissect the role of lignin in HTS, transcriptome data were integrated and genes involved in the lignin biosynthesis pathway in *P. grisea* were identified (Figure S4). Transcriptome and RT‐qPCR results showed that the expression of key lignin biosynthetic genes (*PgPAL1*, *PgC3H*, *PgC4H*, *PgCAD3*) is significantly induced by HTS in wild‐type *P. grisea* plants (Figure 5S5D), consistent with the observed increase in lignin content. These results suggest that lignin accumulation plays a positive role under HTS and that PgZHD18 may protect against the negative effects of high temperature stress by mediating lignin accumulation. To identify the potential lignin biosynthesis‐related targets of PgZHD18, a co‐expression network was constructed based on CIS‐BP, JASPAR, and Plant TFDB databases, and 11 genes in the lignin biosynthesis pathway were identified as potential direct targets of PgZHD18 (Figure S5A). Based on co‐expression network analysis and heatmap of transcriptome data, *PgPAL1*, *PgC4H*, *PgC3H1*, and *PgCAD3* finally showed an upregulated expression pattern under HTS (Figure S5B,D). To verify the regulatory function of PgZHD18 on the candidate target genes, the expression levels of *PgPAL1*, *PgC3H1*, *PgC4H*, and *PgCAD3* in OE‐*PgZHD18* were significantly upregulated compared with the control. In contrast, TRV2:*PgZHD18* significantly downregulated the expression of the above four lignin biosynthesis key genes (Figure S5C), suggesting that PgZHD18 may act as a positive regulator to activate the transcription of core enzyme genes in the lignin biosynthesis pathway. These four genes were all key enzymes in the lignin biosynthesis pathway, and these genes were selected as candidate target genes for further transcriptional regulation studies.

### PgZHD18 activates the expression of *PgPAL1* , *PgC4H* , *PgC3H1* , and *PgCAD3*

To elucidate the regulatory mechanism by which PgZHD18 controls key lignin biosynthetic genes, a series of molecular biology assays to validate its direct transcriptional role was performed. Yeast one‐hybrid (Y1H) assays demonstrated that PgZHD18 binds to the promoter regions of *PgPAL1*, *PgC3H1*, *PgC4H*, and *PgCAD3* (Figure S6). To confirm this interaction in planta, chromatin immunoprecipitation coupled with quantitative PCR (ChIP‐qPCR) was conducted in OE‐*PgZHD18*. The relative enrichment level of GFP group (PgZHD18‐GFP) was significantly higher than that of the IgG control group, indicating that PgZHD18 could directly bind to the promoter regions of *PgPAL1*, *PgC3H1*, *PgC4H*, and *PgCAD3*. After HTS treatment, the enrichment of H3K4me3 at *PgPAL1*, *PgC3H1*, *PgC4H*, and *PgCAD3* binding sites was further increased in the GFP group, indicating that high temperature stress enhanced this DNA binding ability, accompanied by an increase in H3K4me3 enrichment at *PgPAL1*, *PgC3H1*, *PgC4H*, and *PgCAD3* binding sites (Figure 5A). Additionally, dual‐luciferase reporter assays revealed that PgZHD18 strongly activated the expression of luciferase driven by the promoters of these target genes, supporting its function as a transcriptional activator (Figure 5B). To identify the precise binding site, Electrophoretic mobility shift assays (EMSA) were performed. These results indicated that PgZHD18 directly binds to the TAATT cis‐element within the promoters of the lignin biosynthetic genes (Figure 5C). In conclusion, PgZHD18 plays a central role in lignin biosynthesis in *P. grisea* by directly binding to the promoters of key pathway genes (*PgPAL1*, *PgC4H*, *PgC3H1*, and *PgCAD3*) and activating their transcription.

### PgZHD18 interacts with PgWDR5b

**Figure 6:**
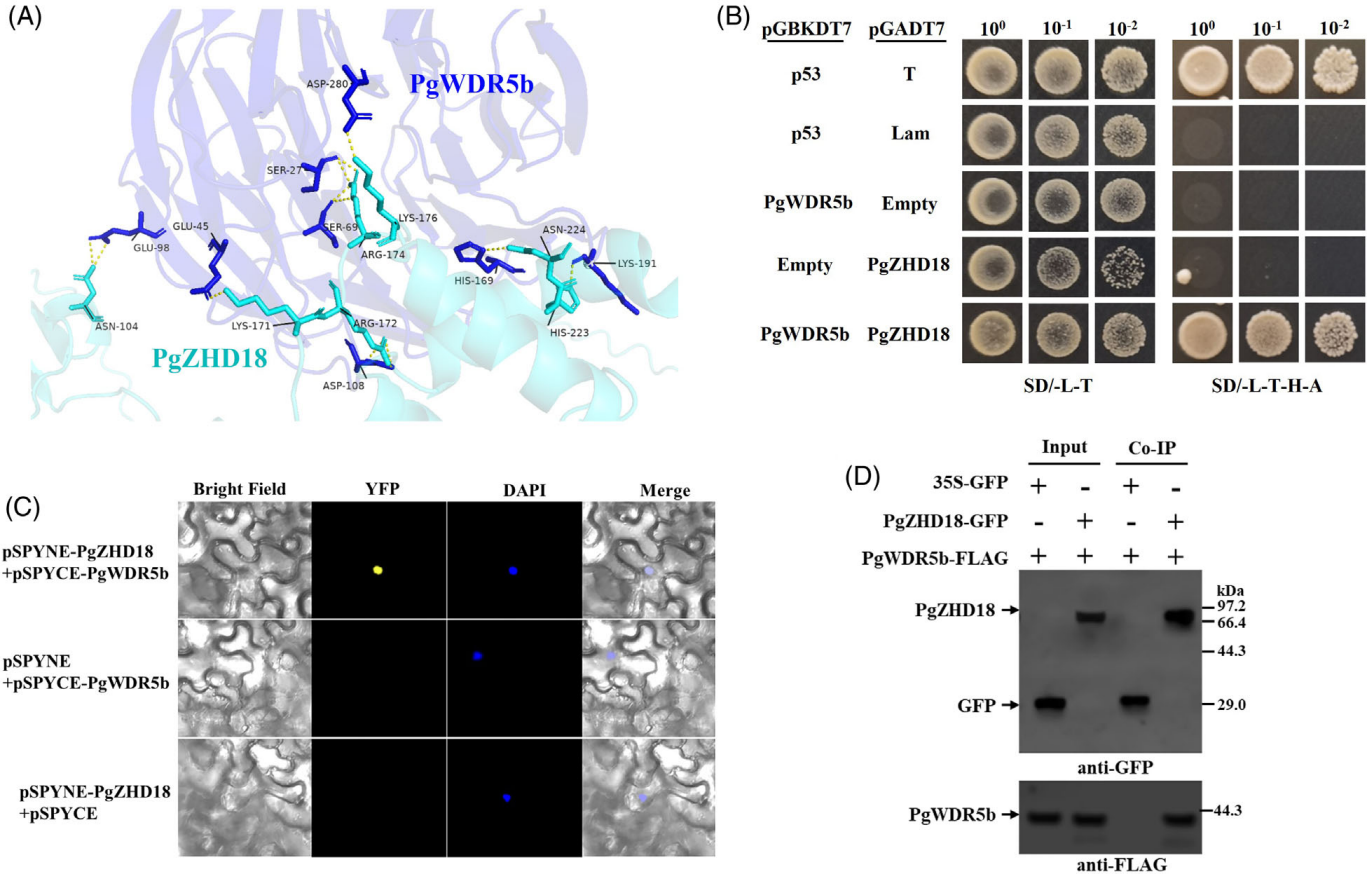
PgZHD18 interacts with PgWDR5b. (A) Alphafold3 predicts the interaction model of PgZHD18 and PgWDR5b, visualized using PyMOL. (B) Y2H indicated that PgZHD18 and PgWDR5b interact with each other. (C) BiFC demonstrated that PgZHD18 and PgWDR5b interacted *in vivo*. pSPYNE‐PgZHD18 + pSPYCE‐PgWDR5b were designated as the experimental groups. pSPYNE + pSPYCE‐PgWDR5b and pSPYNE‐PgZHD18 + pSPYCE served as negative controls. Scale bar = 50 μm. (D) Co‐IP demonstrated that PgZHD18 and PgWDR5b interacted *in vivo*. PgZHD18‐GFP was immunoprecipitated by an antibody against GFP in *N. benthamiana*. Immunoblotting analysis using the FLAG antibody detected the presence of PgWDR5b‐FLAG.

Yeast two‐hybrid (Y2H) screening library was screened using PgZHD18 as the bait protein. PgWDR5b was identified as a candidate interacting protein, which has been confirmed to positively regulate the basal thermotolerance of *P. grisea* (Kong et al., 2025). Protein–protein interaction between PgZHD18 and PgWDR5b was predicted by AlphaFold3, and the spatial interaction model was visualized with PyMOL (Figure 6A). To verify the interaction between PgZHD18 and PgWDR5b, Y2H assays confirmed that PgZHD18 directly interacts with PgWDR5b (Figure 6B). To validate this interaction in planta, bimolecular fluorescence complementation (BiFC) assays were conducted in *N. benthamiana* leaves. Fluorescence signals were observed in the nuclei of cells co‐expressing pSPYNE‐PgZHD18 and pSPYCE‐PgWDR5b, while control combinations (pSPYNE+pSPYCE‐PgWDR5b and pSPYNE‐PgZHD18 + pSPYCE) showed no reconstituted fluorescence (Figure 6C). Furthermore, co‐immunoprecipitation (Co‐IP) experiments were conducted in *N. benthamiana* leaves transiently expressing PgZHD18‐GFP and PgWDR5b‐FLAG. Immunoprecipitation using an anti‐GFP antibody successfully pulled down PgZHD18‐GFP, and subsequent immunoblotting with an anti‐FLAG antibody detected the presence of PgWDR5b‐FLAG in the complex, confirming their physical interaction *in vivo* (Figure 6D). In addition, the expression level of *PgWDR5b* in OE‐*PgZHD18* plants was significantly higher than that in the empty vector control pSAK277 (Figure S7A). However, in TRV2:*PgZHD18* plants, the expression level of *PgWDR5b* was significantly lower than that of the empty vector control TRV2:00 (Figure S7B), and there was a significant positive correlation between PgZHD18 and PgWDR5b expression patterns, suggesting that PgWDR5b transcription can be positively regulated by PgZHD18 expression levels. Taken together, these results provide strong evidence that PgZHD18 and PgWDR5b interact directly to form a protein complex that is likely involved in the HTS response.

### PgZHD18‐PgWDR5b complex enhances H3K4me3 levels of lignin biosynthetic key genes under HTS

**Figure 7:**
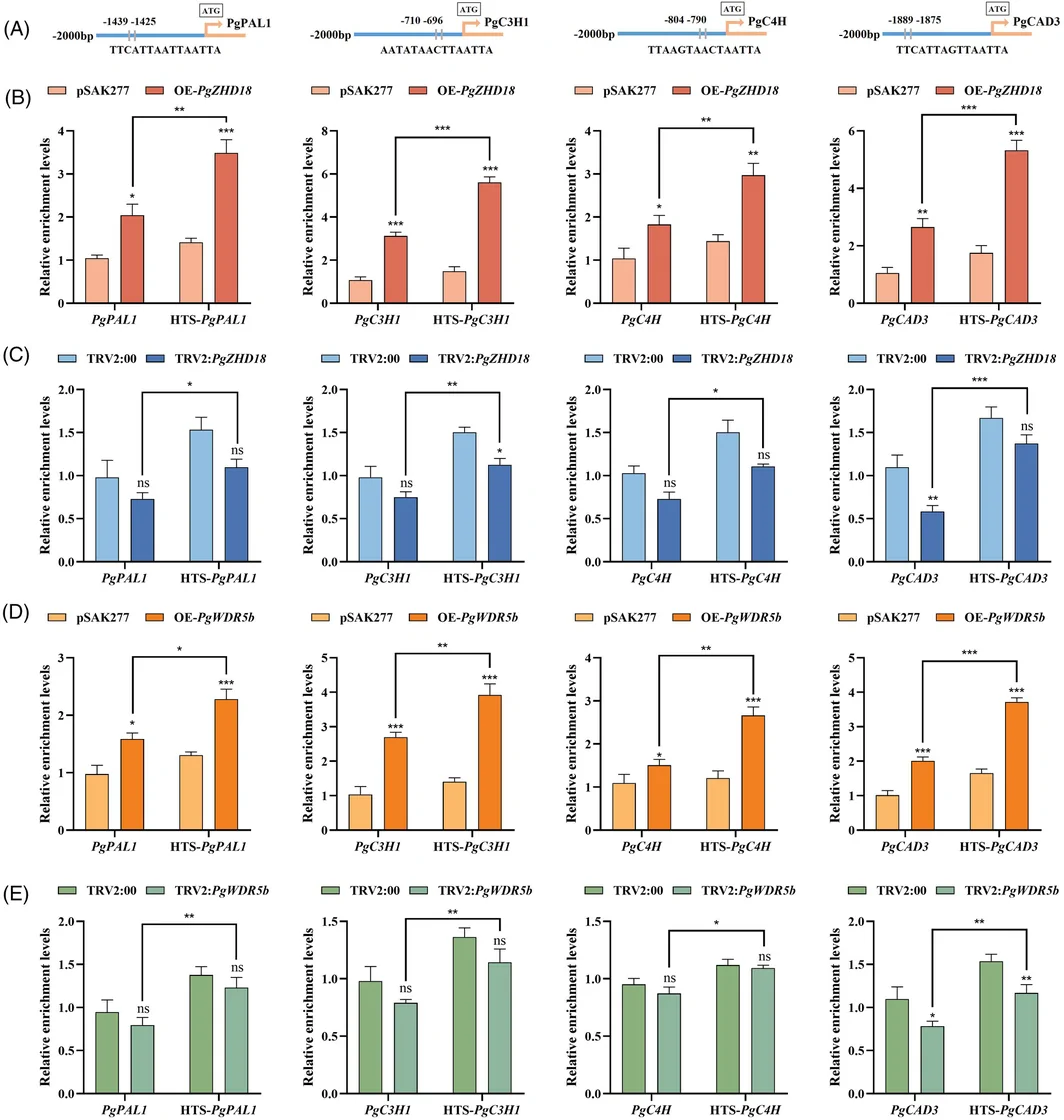
Relative H3K4me3 enrichment levels of target genes in different transgenic lines. (A) Schematic diagram of the sequence‐specific binding sites (TAATT) of PgZHD18 protein and the promoters of *PgPAL1*, *PgC4H*, *PgC3H1*, and *PgCAD3*. (B–C) ChIP‐qPCR analysis determined the relative enrichment levels of H3K4me3 in the *PgPAL1*, *PgC4H*, *PgC3H1*, and *PgCAD3* promoter regions before and after HTS in OE‐*PgZHD18* and TRV2: *PgZHD18* plants. pSAK277 and TRV2:00 were negative controls for OE‐*PgZHD18* and TRV2:*PgZHD18*, respectively. (D, E) ChIP‐qPCR analysis determined the relative enrichment levels of H3K4me3 in the *PgPAL1*, *PgC4H*, *PgC3H1*, and *PgCAD3* promoter regions before and after HTS in OE‐*PgWDR5b* and TRV2:*PgWDR5b* plants. pSAK277 and TRV2:00 were negative controls for OE‐*PgWDR5b* and TRV2:*PgWDR5b*, respectively. Asterisks indicate statistically significant differences (*, *P* < 0.05; **, *P* < 0.01; ***, *P* < 0.001; ns, not significant, Student's *t*‐test). Three biological replicates were set for each assay.

Previous studies have established that WDR5b is a key component in facilitating H3K4me3 modification (Kong et al., 2025; Song et al., 2015). Based on this, a regulatory model has been proposed whereby PgZHD18 binds to the promoter regions of key lignin biosynthetic genes and recruits PgWDR5b to form a protein complex of key lignin biosynthetic genes (*PgPAL1*, *PgC3H1*, *PgC4H*, and *PgCAD3*) and thereby increase H3K4me3 deposition, ultimately improving basal thermotolerance in *P. grisea*. In order to verify this model, ChIP‐qPCR assays in transgenic plants of *PgZHD18* and *PgWDR5b* were performed. Results showed that OE‐*PgZHD18* and OE‐*PgWDR5b* exhibited significantly elevated H3K4me3 enrichment at the promoters of four lignin biosynthetic genes compared to controls, with a further increase following HTS treatment. In contrast, silenced lines TRV2:*PgZHD18* and TRV2:*PgWDR5b* displayed markedly reduced H3K4me3 levels (Figure 7). The results were similar when WT was used as a control (Figure S8). These findings indicate that the PgZHD18‐PgWDR5b protein complex functions in the HTS system to increase the transcriptional activation of the lignin biosynthetic gene promoters by H3K4me3 and thus plays a critical role in *P. grisea* basal thermotolerance.

**Figure 8:**
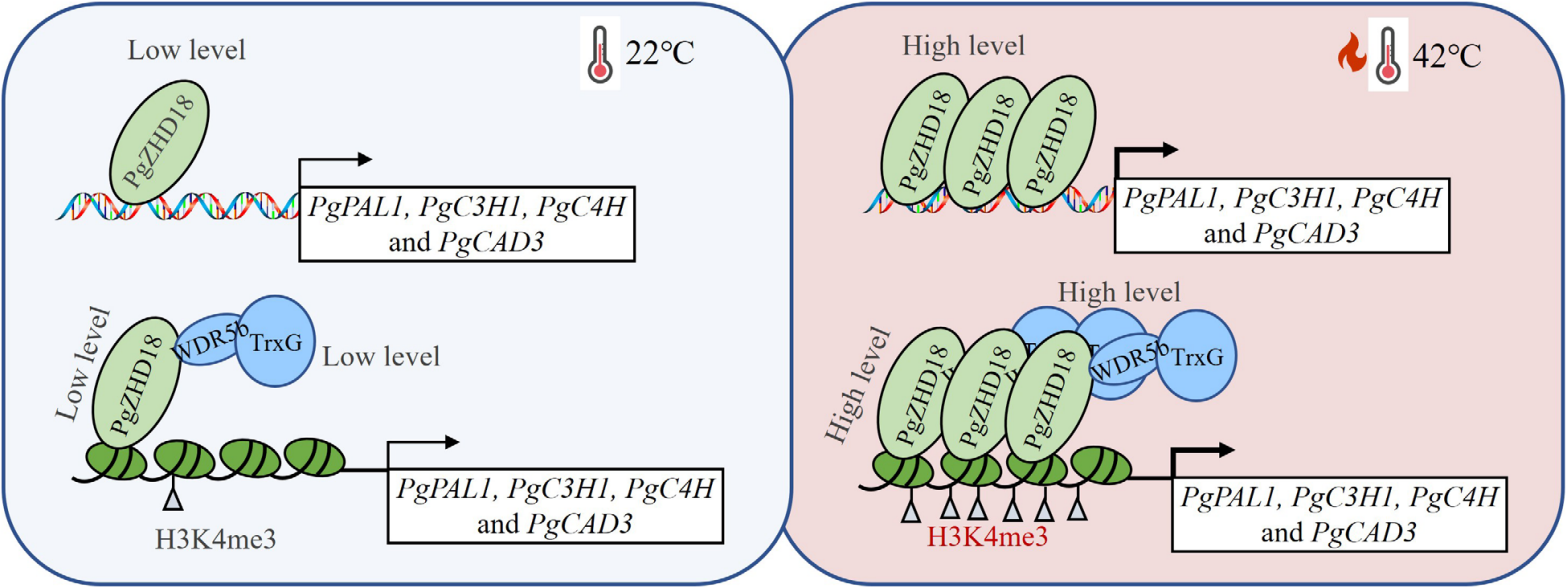
Model for the regulation of *P. grisea* high temperature tolerance by PgZHD18 under HTS. Proposed model illustrating that PgZHD18 directly activates lignin biosynthetic genes and promotes their expression and lignin accumulation through PgWDR5b‐mediated H3K4me3 deposition, thereby enhancing thermotolerance in *P. grisea*. Under normal temperature (22°C), the expression of PgZHD18 and PgWDR5b, as well as the lignin content, were maintained at basal levels. Under HTS (42°C), PgZHD18 directly activated the transcription of lignin biosynthesis genes (*PgPAL1*, *PgC3H1*, *PgC4H*, *PgCAD3*) and interacted with the TrxG protein PgWDR5b to increase H3K4me3 levels, which synergistically enhanced the expression of *PgPAL1*, *PgC3H1*, *PgC4H*, *PgCAD3*, and ultimately improved thermotolerance.

Based on the above data, a working model is proposed (Figure 8). Under normal temperature conditions (22°C), PgZHD18 and PgWDR5b expression remains at a low level. H3K4me3 modifications in promoters of key lignin biosynthesis genes (*PgPAL1*, *PgC3H1*, *PgC4H*, *PgCAD3*) are low, and basal accumulation of lignin is maintained. Under high temperature conditions (42°C), the expression of PgZHD18 and PgWDR5b were significantly induced. PgZHD18 directly activates the transcription of lignin biosynthesis genes. At the same time, the PgZHD18‐PgWDR5b protein complex is stimulated to assemble. This interaction leads to increased levels of H3K4me3 in the target gene promoters and further enhances their transcriptional activation, which in turn increases transcription. Finally, lignin accumulation is enhanced, which synergistically improves the basal thermotolerance of *P. grisea*.

## DISCUSSION

This study elucidates the molecular mechanism by which the PgZHD18‐PgWDR5b module enhances basal thermotolerance in *P. grisea* through the synergistic action of direct transcriptional activation and epigenetic regulation. Our results demonstrate that *PgZHD18*, as a functional transcription factor, directly binds to the promoters of key lignin biosynthetic genes (*PgPAL1*, *PgC3H1*, *PgC4H*, and *PgCAD3*) and activates their transcription under HTS. Meanwhile, PgZHD18 interacts with PgWDR5b to elevate H3K4me3 levels at the promoter regions of these target genes, further amplifying their transcriptional activation, thereby promoting lignin accumulation and basal thermotolerance. These findings provide new insights into the coordinated transcriptional and epigenetic regulatory networks underlying plants' adaptation to HTS.

### PgZHD18 is activated by high temperature and positively regulates basal thermotolerance in *P. grisea*

In this study, expression analysis revealed that HTS significantly induced the transcription of *PgZHD18* (Figure 1A). GUS staining data further confirmed that the *PgZHD18* promoter is specifically activated by high temperature (Figure 1D). Functional characterization demonstrated that OE‐*PgZHD18* plants exhibited improved survival rate under HTS (Figure S2C), along with reduced oxidative damage, higher antioxidant enzyme activities, and enhanced chlorophyll stability (Figure 2). In contrast, TRV2:*PgZHD18* displayed a pronounced heat‐sensitive phenotype (Figure 3). These results indicate that *PgZHD18* expression is thermally activated and that it enhances basal thermotolerance in *P. grisea* by reinforcing the antioxidant defense system. Previous studies have implicated ZHD TFs in abiotic stress responses. In rice, four of the 14 ZHDs proteins can bind to the promoter region of the *DREB1* gene family in response to cold and drought stress (Figueiredo *et al*., 2012), and Arabidopsis *AtZFHD1* contributes to salt stress adaptation (Tran et al., 2007). Recently, the majority of GmZF‐HD genes in soybean showed upregulated expression levels under heat stress treatments. (Rizwan et al., 2025). These findings are consistent with the role of *PgZHD18* uncovered here, suggesting functional conservation among ZHD proteins in plant stress responses. Nevertheless, the upstream signaling mechanisms that activate *ZHD* gene expression under HTS remain unclear and warrant further investigation.

### PgZHD18 enhances basal thermotolerance by directly activating lignin biosynthetic genes

It was observed that there were significant differences in growth and development among different strains at the same growth stage (45 days) under normal conditions (Figure 4A). OE‐*PgZHD18* strains were taller, had longer internodes, and thicker stems compared to the TRV2:*PgZHD18* strains (Figure 4B–D). The relationship between lignin content and plant growth has long been a topic of academic debate: while excessive lignification may restrict cell elongation, site‐specific lignin deposition in vascular tissues and interfascicular fibers is essential for providing mechanical support for stem elongation and maintaining plant upright growth. In this study, *PgZHD18* mainly promoted lignin deposition in vascular bundles and interfascicular fibers of stems, rather than causing ubiquitous lignification across tissues. Accordingly, the growth retardation phenotype of *PgZHD18*‐silenced plants was attributed to reduced lignin content and insufficient mechanical support of stems, which led to stem bending and restricted growth, rather than growth promotion by reduced lignification. This tissue‐specific lignin regulatory pattern is consistent with findings in previous studies (Tang et al., 2023; Wang, Bai, et al., 2025). Thickening growth of the plants' stems is related to the biosynthesis and deposition of lignin (Rogers & Campbell, 2004; Tang et al., 2023). Studies have shown that TFs regulate the expression of lignin biosynthetic genes by binding to their promoters, thereby increasing lignin content. MYB58 in Arabidopsis has been proven to directly bind to the promoters of genes such as *PAL1* and *CAD4* to regulate lignin biosynthesis (Zhou et al., 2009). Rice gene OsMYB30 enhances lignin deposition by activating the expression of *PALs* and *CADs* genes (Liu et al., 2024; Wang, Wang, et al., 2025). Therefore, four key genes involved in lignin biosynthesis (*PgPAL1*, *PgC3H1*, *PgC4H*, and *PgCAD3*) were selected for further verification (Figure S5). It is worth noting that the transcriptome data of lignin biosynthetic genes were not fully consistent with the RT‐qPCR validation results, which reflects the temporal dynamic characteristics of transcriptional cascade regulation. As an upstream transcription factor, *PgZHD18* is rapidly induced by HTS first, and then gradually activates the transcription of downstream lignin biosynthetic genes through regulatory cascades. This leads to a time lag between the expression peak of *PgZHD18* and the upregulation of downstream structural genes, which explains the partial inconsistency between transcriptome sampling at fixed time points and the detailed time‐course RT‐qPCR data. Y1H experiment demonstrated that PgZHD18 could bind to the promoters of *PgPAL1*, *PgC3H1*, *PgC4H*, and *PgCAD3* (Figure S6). ChIP‐qPCR and dual luciferase assays revealed that PgZHD18 could activate the transcription of these genes and was significantly enriched after HTS treatment (Figure 5A,B). Moreover, EMSA experiments indicated that PgZHD18 could bind to the TAATT motifs of these gene promoters (Figure 5C).

Based on the combined evidence of PgZHD18 directly activating lignin biosynthetic genes, increased lignin content under normal conditions in OE‐*PgZHD18* plants, upregulation of lignin biosynthetic genes under HTS, and enhanced thermotolerance and reduced stem lodging in OE‐*PgZHD18* plants under HTS, it is likely that PgZHD18 enhances basal thermotolerance by promoting lignin biosynthesis. Studies have shown that the accumulation of lignin under HTS helps maintain cell structural integrity and reduces water loss, thereby enhancing the thermotolerance of plants (Dong & Lin, 2021; Guo et al., 2023; Han et al., 2022). In bamboo, PeCDPK32 regulates the activity of peroxidase PePRX2 to promote lignin deposition, thereby enhancing the mechanical strength of the stem and the stress resistance of the plant (Li et al., 2025). This provides strong support for the mechanism by which PgZHD18 regulates lignin biosynthesis to enhance basal thermotolerance. We attempted to perform histological staining of lignin in HTS‐treated seedlings, but found that the stems were too small and fragile to obtain high‐quality cross sections suitable for quantitative analysis. This technical limitation could be addressed in future studies using older plants at later developmental stages. It is worth noting that plants usually respond to stress by coordinating multiple metabolic pathways. How the deposition of lignin interacts with pathways such as ROS clearance and hormone signaling still requires further exploration.

### PgZHD18‐PgWDR5b interaction increases H3K4me3 levels of lignin biosynthetic key genes

Interactions between TFs and epigenetic regulators in plants enable the specific recruitment of histone‐modifying enzymes to target gene loci, allowing precise control of gene expression (Zhu et al., 2023). It has been previously established by our group that PgWDR5b, a component of the COMPASS‐like complex, positively regulates basal thermotolerance in *P. grisea* (Kong et al., 2025). In this study, AlphaFold3 predicted a physical interaction between PgZHD18 and PgWDR5b (Figure 6A), which was subsequently confirmed through yeast Y2H, BiFC, and Co‐IP assays (Figure 6B–D). Studies of plant stress responses have shown that HTS can induce H3K4me3 deposition at the promoters of stress responsive genes (He et al., 2025; Huang et al., 2019). Given that WDR5 primarily facilitates H3K4me3 modification on downstream genes (Song et al., 2015), ChIP‐qPCR assays were conducted using transgenic lines with altered expression of PgZHD18 or PgWDR5b. Results revealed significantly elevated H3K4me3 levels at the promoters of lignin biosynthetic genes in overexpression lines, with a further increase under HTS. In contrast, silenced lines showed markedly reduced H3K4me3 enrichment (Figure 7). These findings indicate that the PgZHD18‐PgWDR5b protein complex enhances H3K4me3 modification of lignin biosynthetic genes under HTS conditions. However, important questions remain regarding how the dynamics and reversibility of the H3K4me3 epigenetic mark are finely tuned in response to environmental cues, and whether additional histone modifications also contribute to this regulatory mechanism. These questions represent key directions for future research.

## MATERIALS AND METHODS

### Plants materials and HTS treatments

*P. grisea* and *Nicotiana benthamiana* plants were cultivated in a greenhouse at 22°C under a 16 h light /8 h dark photoperiod, with a light intensity of 60–65 μmol photons·m^−2^·s^−1^. *P. grise*a seeds were germinated on MS medium for 10 days before being transplanted into pots containing a mixture of perlite, vermiculite, and compost. Plants at the 5–6 true‐leaf stage were transferred to a controlled climate chamber (MGC‐150BP, HuaLin) and subjected to HTS treatment at 42°C. Each group was treated with at least three transgenic lines of 20 plants per line. During the treatment, the soil was regularly watered to ensure an adequate moisture content was maintained. The intact plants treated at 42°C for 0 h, 24 h, 48 h, and recovered at 22°C for 48 h were collected for physiological indices and survival rate statistics. The survival criterion was that plants could continue to grow and develop, remain green (not browning), and have straight, non‐wilting stems.

### RNA‐seq analysis

The transcriptome sequencing data reported in the present study has been deposited in the National Center for Biotechnology Information (NCBI) database under project number PRJNA1171172. Sequencing was performed on the Illumina NovaSeq 6000 platform with three biological replicates per treatment. The *Physalis grisea* v1.0 genome was used as the reference for read alignment. Differential gene expression was analyzed using DESeq2, with screening criteria set as FDR <0.05 and |log2 fold change| >1. The specific analysis method has been published in The Plant Journal. (Jing et al., 2025).

### DNA and RNA analysis and RT‐qPCR

Total RNA was extracted using the YALEPIC Plant RNA Fast Isolation Kit (PLUS) (YALEPIC, Jiangsu, China) according to the manufacturer's instructions. The samples were stored at 80°C in an ultralow temperature refrigerator. Genomic DNA was isolated with the YALEPIC Plant Genomic DNA Isolation Kit (PLUS) (YALEPIC, Jiangsu, China). cDNA was synthesized using the M5 First Strand cDNA Synthesis Kit (Mei5bio, Beijing, China). Quantitative real‐time PCR (RT‐qPCR) was performed using the 2^−▵▵Ct^ method (Rao et al., 2013). *PgACTIN* (*Phygri08g031070*) was used as an internal reference to normalize quantitative fluorescence data (Zhang et al., 2024). Intact plants were collected for HTS treatment for RT‐qPCR. Asterisks indicate statistically significant differences (*, *P* < 0.05; **, *P* < 0.01; ***, *P* < 0.001; ns, not significant, Student's *t*‐test). Three biological replicates were set for each assay.

### Overexpression and virus‐induced gene silencing (VIGS) in *P. grisea*

Overexpression and VIGS assays were performed as previously described (Jing et al., 2025; Zhang et al., 2024). The pSAK277 vector was used for the overexpression of *PgZHD18* via genetic transformation in *P. grisea*. A 329 bp‐specific fragment of *PgZHD18* was amplified using the 2× SevenStar PFU PCR MasterMix (Seven/Abcells, Beijing, China) and subsequently inserted into the pTRV2 vector for VIGS.

### Subcellular localization

The PgZHD18 coding sequence was fused to yellow fluorescent protein (YFP) in the pYFP101 vector to generate the YFP‐PgZHD18 construct. The fusion plasmid was transformed into *A. tumefaciens* strain (GV3101) and infiltrated into leaves of *N. benthamiana*. YFP fluorescence was observed using confocal laser scanning microscopy.

### β‐Glucuronidase staining analysis

β‐Glucuronidase (GUS) staining as previously described (Kong et al., 2025). In this study, the promoter (2000 bp upstream) of *PgZHD18* was constructed in the GUS reporter gene. Treatment of injectable *Nicotiana benthamiana* using the injection method. Transcription activation verification (TF: gene promoter = 1:1) treatment of *N. benthamiana* plants involved cutting the leaves and subjecting them to vacuum extraction. CaMV35S::GUS was used as a positive control. GUS and Pro*PgZHD18*::GUS (22°C) treated at 22°C for 24 h were used as negative controls. Pro*PgZHD18*::GUS + HTS (42°C) treated at 42°C for 24 h were used as the experimental group. Three biological replicates were set. GUS staining was performed using the GUSBlue Kit (Huayueyang, Beijing, China) following the manufacturer's protocol.

### Dual‐luciferase assay and EMSA

The promoters of *PgPAL1*, *PgC3H1*, *PgC4H*, and *PgCAD3* were cloned into the pGreenII 0800‐LUC vector via homologous recombination. The resulting recombinant plasmids, LUC‐*ProPgPAL1*, LUC‐*ProPgC3H1*, LUC‐*ProPgC4H*, and LUC‐*ProPgCAD3*, were transformed into *A. tumefaciens* strain (GV3101‐pSoup) to serve as reporters. The full‐length coding sequence of *PgZHD18* was inserted into the pSAK277 vector to generate the effector construct OE‐*PgZHD18*, which was then introduced into *A. tumefaciens* strain (GV3101). A mixture of the effector and reporter strains at a 9:1 ratio was infiltrated into leaves of 5‐week‐old *N. benthamiana* plants. A control was prepared by co‐infiltrating the reporter with a strain carrying the empty 35S vector. After infiltration, plants were kept in darkness for 12 h and then under normal light for 2 days. Firefly luciferase (LUC) and Renilla luciferase (REN) activities were measured using the GloMax® Navigator System (Promega, USA). The ratio of LUC to REN luminescence was used to calculate relative promoter activity. Asterisks indicate statistically significant differences (*, *P* < 0.05; **, *P* < 0.01; ***, *P* < 0.001; ns, not significant, Student's *t*‐test). Three biological replicates were set for each assay. The full‐length PgZHD18 protein was fused with pGEX‐6p‐1 to construct a recombinant plasmid and expressed in the *E. coli* BL21 strain. The protein was subsequently purified using GST agarose affinity chromatography (Xu et al., 2021). For the EMSA, *PgPAL1*, *PgC3H1*, *PgC4H*, and *PgCAD3* promoter fragments containing TAATT ciselements were biotin‐labeled to serve as probes, while the same unlabeled DNA fragments were utilized as competitors in the assay. EMSA was performed according to the manufacturer's instructions using a LightShift chemiluminescent EMSA kit (Thermo Fisher Scientific).

### Physiological index measurements

For the determination of antioxidant enzyme content, 0.1 g of fresh leaves were taken, froze them quickly in liquid nitrogen, and then grinded them into powder in a mortar. The powder was transferred to a centrifuge tube and 3 ml of PBS was added (50 mM, pH = 7.0) (containing 0.1% polyvinylpyrrolidone and 5 mM dithiothreitol). It was centrifuged for 20 min at 4°C and 15 000 rpm. The supernatant was collected, which is the enzyme solution to be measured subsequently. For the determination of SOD content, 100 μl of the enzyme solution was taken, 2 ml of PBS (50 mM, pH = 7.8), 0.3 ml of methionine (130 mM), 0.3 ml of NBT (750 μM), and 10 μl of riboflavin (7 mM) were added. After mixing, irradiate under a 15 W fluorescent lamp for 3 min, then measure the absorbance at 560 nm. Analyzed SOD content using previous methods (Giannopolitis & Ries, 1977). For the determination of POD content, the guaiacol oxidation method was used (Killi et al., 2020). For the determination of CAT content, the method used by previous researchers was employed (Pang et al., 2024). For MDA content assay, NBT and DAB staining methods were performed as described previously (Kong et al., 2025). For chlorophyll, content determination method was performed as described previously (Kong et al., 2025). Asterisks indicate statistically significant differences (*, *P* < 0.05; **, *P* < 0.01; ***, *P* < 0.001; ns, not significant, Student's *t*‐test). Three biological replicates were set for each assay.

### Determination of lignin content

Lignin was extracted from the stems of *P. grisea* following the protocol provided with the Lignin Content Kit (Acetylation Method) (AIDISHENG, Jiangsu, China). For HTS‐treated samples, stems were collected to determine lignin content after 0 h, 24 h, and 48 h of treatment at 42°C and 48 h of recovery at 22°C.

The stem segments (from the third internode of the 45 days of different transgenic lines of water spinach) underwent wax embedding. The resulting tissue sections were subjected to toluidine blue staining for lignin observation (O'brien, 1964). The lignin staining is the blue area.

### Yeast hybrid assays

For Y1H assays, the full length of the promoters of *PgPAL1*, *PgC3H1*, *PgC4H*, and *PgCAD3* was amplified and cloned into the pAbAi bait vector. The full‐length PgZHD18 sequence was inserted into pGADT7. The bait construct was linearized with BstBI and integrated into the yeast strain Y1H Gold. Positive colonies were selected on SD/‐Ura medium. The prey plasmid was transformed into the bait strain and interactions were tested on SD/‐Leu medium supplemented with aureobasidin A (AbA). The empty pGADT7 vector was used as a negative control.

For Y2H assays, pGADT7‐PgZHD18 and pGBKDT7‐PgWDR5b vectors were cloned separately. Co‐transformed yeast cells were plated on SD/‐Trp/‐His and then SD/‐Trp/‐Leu/‐His/‐Ade dropout media for interaction screening. pGBKT7‐p53 + pGADT7‐T and pGBKT7‐p53 + pGADT7‐Lam were used as positive and negative controls, respectively.

### ChIP‐qPCR analysis

ChIP was performed using chromatin immunoprecipitation (ChIP) Kit for Plant (GeneCreate, Wuhan, China). Plants overexpressing ZHD18‐GFP (OE‐PgZHD18) were used as experimental materials, and IgG immunoprecipitation was set as a negative control. Plants grown to the sixth 5–6 leaf stage were divided into two groups: one group was treated with normal temperature (control group, labeled *PgPAL1*, *PgC3H1*, *PgC4H*, and *PgCAD3*), and the other group was treated with high temperature stress (HTS) (treatment group, labeled HTS‐*PgPAL1*, HTS‐*PgC3H1*, HTS‐*PgC4H*, and HTS‐PgCAD3). After the nucleus was extracted from the treated stem segments, chromatin was fragmented to 200–500 bp by ultrasonic fragmentation. Immunoprecipitated purified DNA fragments were obtained by addition of anti‐GFP (Abmart, M20004) antibody (enriched for PgZHD18‐bound DNA fragments) and IgG antibody (nonspecific control), respectively. The enriched DNA was used as a template to design specific primers (Table S1) for the promoter region of the *PgPAL1*, *PgC3H1*, *PgC4H*, and *PgCAD3* genes, and qPCR was performed. Normalization was performed according to the enrichment level of the IgG control group.

Different genetically modified plants were used as experimental materials, and plants carrying empty vectors pSAK277, TRV2:00 were used as controls. Anti‐H3K4me3 (ABclonal A2357)‐specific antibody was added for immunoprecipitation to detect the enrichment of H3K4me3 on *PgPAL1*, *PgC3H1*, *PgC4H*, and *PgCAD3* promoters. The enrichment of H3K4me3 in the control group was used as the basis for normalization. Relative enrichment levels were calculated using the 2^−▵▵Ct^ method to reflect the binding of PgZHD18 to *PgPAL1*, *PgC3H1*, *PgC4H*, and *PgCAD3* promoters and the enrichment level of H3K4me3 modifications. Asterisks indicate statistically significant differences (*, *P* < 0.05; **, *P* < 0.01; ***, *P* < 0.001; ns, not significant, Student's *t*‐test). Three biological replicates were set for each assay.

### Co‐immunoprecipitation (co‐IP) assay

Three combinations of constructs (PgZHD18‐GFP + PgWDR5b‐FLAG; control sets with single tags) were transiently co‐expressed in 4‐week‐old *N. benthamiana* leaves. After 48 h, total protein was extracted using NP‐40 lysis buffer (Beyotime, P0013F) and incubated with anti‐GFP antibody (Proteintech, 50 430‐2‐AP) for 1 h at 4°C. The mixture was supplemented with 1× protease inhibitor cocktail (Abmart) and MG132, gently agitated, and incubated overnight at 4°C. Then, 50 μL of protein A/G agarose beads (Yeasen) was added and incubated for 2 h. Immunoprecipitates were washed with PBS and boiled in 1× SDS loading buffer. Proteins were detected by immunoblotting using anti‐GFP (Abmart, M20004) and anti‐FLAG (Abmart, M20008) antibodies.

### Bimolecular fluorescence complementation (BiFC) assay

PgZHD18 and PgWDR5b were fused to the N‐terminal (pSPYNE) and C‐terminal (pSPYCE) fragments of YFP, respectively. Constructs were introduced into *A. tumefaciens* GV3101 and infiltrated into *N. benthamiana* leaves in the following combinations: pSPYNE‐PgZHD18 + pSPYCE‐PgWDR5b, pSPYNE + pSPYCE‐PgWDR5b, and pSPYNE‐PgZHD18 + pSPYCE. YFP fluorescence was observed using a DM4B fluorescence microscope (Leica, Germany) with an external light source (EL6000; Leica). Three replicates were performed, with at least three leaves observed per replicate.

### Bioinformatics analysis

A phylogenetic tree was constructed with MEGA11 software using the neighbor‐joining method. Heat maps are drawn using Tbtools (Chen et al., 2023). Protein interaction predictions were made using AlphaFold 3. Spatial interaction model diagrams were drawn using PyMOL software.

### Data analysis

Transcriptome data are available in GenBank under accession number PRJNA1171172. Data were analyzed and visualized using GraphPad Prism 9.0. Phylogenetic trees were constructed using MEGA11. Heatmaps and bioinformatics analysis were performed using TBtools v1.120. Student's *t*‐test was used for pairwise comparisons between two groups. All experiments were repeated at least three times independently. All primers listed are detailed in Table S1.

## AUTHOR CONTRIBUTIONS

HL and YY conceptualized and designed the research. HL and GK performed major experiments. YZ, YR, and JYN performed overexpression transformation and VIGS silencing injection. HL, XZ, and GK performed phenotypic observations and statistics. HL and QS analyzed the data. HL, QS, and YY wrote the manuscript. All authors read and approved the contents of this paper.

## CONFLICT OF INTEREST

The authors declare no competing interest.

## Supporting information


**Figure S1.** Identification *PgZHD18* of *P. grisea*. (A) Phylogenetic analysis of *PgZHD18*. Different colored dots represent the corresponding species (purple, *Solanum lycopersicum*; green, *Arabidopsis thaliana*; pink, *P. grisea*), I–VI represent different subfamilies. The red asterisk indicates the homologous protein of PgZHD18 in *P. grisea*. (B) Heatmap based on RNA‐seq data shows the transcript levels (expressed as log_2_FC) of *PgZHDs* in *P. grisea* after HTS at 42°C for 12 h, 24 h, 36 h, and 48 h. The gene id marked in the red rectangle is *PgZHD18*.
**Figure S2.** Identification of OE‐*PgZHD18*‐positive plants, survival rate of HTS and analysis of lignin content. (A) Identification of transgenic positive plants by gel electrophoresis. (B) Expression levels of the target genes in the transgenic plants were detected by RT‐qPCR. (C) Survival rate of transgenic plants treated with HTS for 0 h, 24 h, 48 h, and recovery for 48 h. All three independent OE‐*PgZHD18* lines (#1, #2, #3) were included in the statistical analysis. Each line contained 20 plants, and the experiment was repeated three times independently. pSAK277 were negative controls for OE‐*PgZHD18*. (D) Lignin content in pSAK277 and OE‐*PgZHD18* plants. Asterisks indicate statistically significant differences (*, *P* < 0.05; **, *P* < 0.01; ***, *P* < 0.001; ns, not significant, Student's *t*‐test). Three biological replicates were set for each assay.
**Figure S3.** Identification of TRV2:*PgZHD18*‐positive plants, survival rate of HTS and analysis of lignin content. (A) Identification of transgenic positive plants by gel electrophoresis. (B) Expression levels of the target genes in the transgenic plants were detected by RT‐qPCR. (C) Survival rate of transgenic plants treated with HTS for 0 h, 24 h, 48 h, and recovery for 48 h. All three independent TRV2:*PgZHD18* lines (#1, #2, #3) were included in the statistical analysis. Each line contained 20 plants, and the experiment was repeated three times independently. TRV2:00 were negative controls for TRV2:*PgZHD18*. (D) Lignin content in TRV2:00 and TRV2:*PgZHD18* plants. Asterisks indicate statistically significant differences (*, *P* < 0.05; **, *P* < 0.01; ***, *P* < 0.001; ns, not significant, Student's *t*‐test). Three biological replicates were set for each assay.
**Figure S4.** Lignin biosynthesis pathway and related gene expression under HTS in *P. grisea*. Identify the genes involved in the lignin biosynthesis pathway, integrate the transcriptome data and represent the expression patterns of the genes coding for the key enzymes in this pathway by means of heatmaps.
**Figure S5.** Targeted relationship and expression pattern of *PgZHD18* with genes related to the lignin biosynthesis pathway. (A) Targeted relationship between PgZHD18 and genes related to the lignin biosynthesis pathway. Nodes represent genes, and the lines indicate co‐expression associations. The pink nodes represent genes that co‐express with *PgZHD18* and are involved in lignin synthesis, while the green nodes represent other genes. (B) Heatmaps based on RNA‐seq data show transcript levels (expressed as log_2_FC) of 11 lignin biosynthesis‐related genes that can be co‐expressed with PgZHD18 at 42°C for 12 h, 24 h, 36 h, and 48 h. The target gene is marked in the red rectangle. (C) Expression levels of *PgPAL1*, *PgC3H1*, *PgC4H*, and *PgCAD3* in OE‐*PgZHD18* and TRV2:*PgZHD18* under normal conditions. (D) Expression level of *PgPAL1*, *PgC3H1*, *PgC4H*, and *PgCAD3* after HTS treatment by RT‐qPCR. Asterisks indicate statistically significant differences (*, *P* < 0.05; **, *P* < 0.01; ***, *P* < 0.001; ns, not significant, Student's *t*‐test). Three biological replicates were set for each assay.
**Figure S6.** Y1H experiment demonstrated that PgZHD18 can bind to the promoter of the target genes. Y1H assay indicates that PgZHD18 can bind to the promoters of *PgPAL1*, *PgC3H1*, *PgC4H*, and *PgCAD3*. SD/‐Leu is a yeast medium lacking leucine. Yeast strains co‐transformed with AD‐PgZHD18 and *PgPAL1*, *PgC3H1*, *PgC4H*, *PgCAD3* promoters with the full‐length were able to grow normally on AbA (Aureobasidin A; 400 ng·ml^−1^).
**Figure S7.** Expression levels of *PgWDR5b* in OE‐*PgZHD18* and TRV2:*PgZHD18*. (A) Expression level of *PgWDR5b* in OE‐*PgZHD18*. (B) Expression level of *PgWDR5b* in TRV2:*PgZHD18*. Asterisks indicate statistically significant differences (*, *P* < 0.05; **, *P* < 0.01; ***, *P* < 0.001; ns, not significant, Student's *t*‐test). Three biological replicates were set for each assay.
**Figure S8.** Relative H3K4me3 enrichment levels of target genes in different transgenic lines. (A) Schematic diagram of the sequence‐specific binding sites (TAATT) of PgZHD18 protein and the promoters of *PgPAL1*, *PgC4H*, *PgC3H1*, and *PgCAD3*. (B, C) ChIP‐qPCR analysis determined the relative enrichment levels of H3K4me3 in the *PgPAL1*, *PgC4H*, *PgC3H1*, and *PgCAD3* promoter regions before and after HTS in OE‐*PgZHD18* and TRV2: *PgZHD18* plants. WT was negative controls for OE‐*PgZHD18* and TRV2:*PgZHD18*. (D, E) ChIP‐qPCR analysis determined the relative enrichment levels of H3K4me3 in the *PgPAL1*, *PgC4H*, *PgC3H1*, and *PgCAD3* promoter regions before and after HTS in OE‐*PgWDR5b* and TRV2:*PgWDR5b* plants. WT was negative controls for OE‐*PgWDR5b* and TRV2:*PgWDR5b*. Asterisks indicate statistically significant differences (*, *P* < 0.05; **, *P* < 0.01; ***, *P* < 0.001; ns, not significant, Student's *t*‐test). Three biological replicates were set for each assay.

**Table S1.** Nucleotide sequences of all primers used in this study.

## Data Availability

The data that supports the findings of this study are available in the supplementary material of this article. In addition, the transcriptome sequencing data reported in the present study are openly available at https://www.ncbi.nim.nih.gov/bioproject/PRJNA1171172/.
