# Supplementary material for: PgZHD18‐PgWDR5b increases basal thermotolerance in Physalis grisea by lignin biosynthesis dependent on H3K4me3
Source: Plant J. 2026 Sep 21;127(6):e71131. doi: 10.1111/tpj.71131 (PMC13591493; doi:10.1111/tpj.71131)
Supplement: Supplementary file 1 [file TPJ-127-0-s001.docx]

**Supplemental Figure 1** Identification *PgZHD18* of *P. grisea*.

(A) Phylogenetic analysis of *PgZHD18*. Different colored dots represent the corresponding species (purple, *Solanum lycopersicum*; green, *Arabidopsis thaliana*; pink, *P. grisea*), Ⅰ-Ⅵ represent different subfamilies. The red asterisk indicates the homologous protein of PgZHD18 in *P. grisea*. (B) Heatmap based on RNA-seq data shows the transcript levels (expressed as log_2_FC) of *PgZHDs* in *P. grisea* after HTS at 42℃ for 12 h, 24 h, 36 h, and 48 h. The gene id marked in the red rectangle is *PgZHD18*.

**Supplemental Figure 2** Identification of OE-*PgZHD18* positive plants, survival rate of HTS and analysis of lignin content.

(A) Identification of transgenic positive plants by gel electrophoresis. (B) Expression levels of the target genes in the transgenic plants were detected by RT-qPCR. (C) Survival rate of transgenic plants treated with HTS for 0 h, 24 h, 48 h and recovery for 48 h. All three independent OE-*PgZHD18* lines (#1, #2, #3) were included in the statistical analysis. Each line contained 20 plants, and the experiment was repeated three times independently. pSAK277 were negative controls for OE-*PgZHD18*. (D) Lignin content in pSAK277 and OE-*PgZHD18* plants. Asterisks indicate statistically significant differences (*, *P* < 0.05; **, *P* < 0.01; *, *P* < 0.001; ns, not significant, Student’s *t*-test). Three biological replicates were set for each assay.

**Supplemental Figure 3** Identification of TRV2:*PgZHD18* positive plants, survival rate of HTS and analysis of lignin content.

(A) Identification of transgenic positive plants by gel electrophoresis. (B) Expression levels of the target genes in the transgenic plants were detected by RT-qPCR. (C) Survival rate of transgenic plants treated with HTS for 0 h, 24 h, 48 h and recovery for 48 h. All three independent TRV2:*PgZHD18* lines (#1, #2, #3) were included in the statistical analysis. Each line contained 20 plants, and the experiment was repeated three times independently. TRV2:00 were negative controls for TRV2:*PgZHD18*. (D) Lignin content in TRV2:00 and TRV2:*PgZHD18* plants. Asterisks indicate statistically significant differences (*, *P* < 0.05; **, *P* < 0.01; *, *P* < 0.001; ns, not significant, Student’s *t*-test). Three biological replicates were set for each assay.

**Supplemental Figure 4** Lignin biosynthesis pathway and related gene expression under HTS in *P. grisea*.

Identify the genes involved in the lignin biosynthesis pathway, integrate the transcriptome data and represent the expression patterns of the genes coding for the key enzymes in this pathway by means of heatmaps.

**Supplemental Figure 5** Targeted relationship and expression pattern of *PgZHD18* with genes related to the lignin biosynthesis pathway.

(A) Targeted relationship between PgZHD18 and genes related to the lignin biosynthesis pathway. Nodes represent genes, and the lines indicate co-expression associations. The pink nodes represent genes that co-express with *PgZHD18* and are involved in lignin synthesis, while the green nodes represent other genes. (B) Heatmaps based on RNA-seq data show transcript levels (expressed as log_2_FC) of 11 lignin biosynthesis related genes that can be co-expressed with PgZHD18 at 42℃ for 12 h, 24 h, 36 h and 48 h. The target gene is marked in the red rectangle. (C) Expression levels of *PgPAL1*, *PgC3H1*, *PgC4H* and *PgCAD3* in OE-*PgZHD18* and TRV2:*PgZHD18* under normal conditions. (D) Expression level of *PgPAL1*, *PgC3H1*, *PgC4H* and *PgCAD3* after HTS treatment by RT-qPCR. Asterisks indicate statistically significant differences (*, *P* < 0.05; **, *P* < 0.01; ***, *P* < 0.001; ns, not significant, Student’s *t*-test). Three biological replicates were set for each assay.

**Supplemental Figure 6** Y1H experiment demonstrated that PgZHD18 can bind to the promoter of the target genes.

Y1H assay indicates that PgZHD18 can bind to the promoters of *PgPAL1*, *PgC3H1*, *PgC4H* and *PgCAD3*. SD/-Leu is a yeast medium lacking leucine. Yeast strains co-transformed with AD-PgZHD18 and *PgPAL1*, *PgC3H1*, *PgC4H*, *PgCAD3* promoters with the full-length were able to grow normally on AbA (Aureobasidin A; 400 ng·ml^-1^).

**Supplemental Figure 7** Expression levels of *PgWDR5b* in OE-*PgZHD18* and TRV2:*PgZHD18*.

(A) Expression level of *PgWDR5b* in OE-*PgZHD18*. (B) Expression level of *PgWDR5b* in TRV2:*PgZHD18.* Asterisks indicate statistically significant differences (*, *P* < 0.05; **, *P* < 0.01; ***, *P* < 0.001; ns, not significant, Student’s *t*-test). Three biological replicates were set for each assay.

**Supplemental Figure 8** Relative H3K4me3 enrichment levels of target genes in different transgenic lines.

(A) Schematic diagram of the sequence-specific binding sites (TAATT) of PgZHD18 protein and the promoters of *PgPAL1*, *PgC4H*, *PgC3H1* and *PgCAD3*. (B-C) ChIP-qPCR analysis determined the relative enrichment levels of H3K4me3 in the *PgPAL1*, *PgC4H*, *PgC3H1* and *PgCAD3* promoter regions before and after HTS in OE-*PgZHD18* and TRV2: *PgZHD18* plants. WT was negative controls for OE-*PgZHD18* and TRV2:*PgZHD18*. (D-E) ChIP-qPCR analysis determined the relative enrichment levels of H3K4me3 in the *PgPAL1*, *PgC4H*, *PgC3H1* and *PgCAD3* promoter regions before and after HTS in OE-*PgWDR5b* and TRV2:*PgWDR5b* plants. WT was negative controls for OE-*PgWDR5b* and TRV2:*PgWDR5b*. Asterisks indicate statistically significant differences (*, *P* < 0.05; **, *P* < 0.01; ***, *P* < 0.001; ns, not significant, Student’s *t*-test). Three biological replicates were set for each assay.
